# A causal role for the cerebellum in semantic integration: a transcranial magnetic stimulation study

**DOI:** 10.1038/s41598-020-75287-z

**Published:** 2020-10-23

**Authors:** Daniele Gatti, Floris Van Vugt, Tomaso Vecchi

**Affiliations:** 1 Department of Brain and Behavioral Sciences, University of Pavia, via Bassi 21, 27100 Pavia, Italy; 2 Psychology Department, University of Montreal, Montreal, H3A1G1 Canada; 3 IRCCS Mondino Foundation, 27100 Pavia, Italy

**Keywords:** Neuroscience, Psychology

## Abstract

Mounting evidence suggests that the cerebellum, a structure previously linked to motor function, is also involved in a wide range of non-motor processes. It has been proposed that the cerebellum performs the same computational processes in both motor and non-motor domains. Within motor functions, the cerebellum is involved in the integration of signals from multiple systems. Here we hypothesized that cerebellum may be involved in integration within semantic memory as well. Specifically, understanding a noun-adjective combination (e.g. red apple) requires combining the meaning of the adjective (red) with the meaning of the noun (apple). In two experiments, participants were asked to judge whether noun-adjective word-pairs were semantically related (e.g., red apple) or not (e.g., lucky milk) while online transcranial magnetic stimulation (TMS) was administered over the right cerebellum or over a control site (vertex in Experiment 1 and visual cortex in Experiment 2). Cerebellar TMS caused a decrease in participants’ accuracy for related word-pairs while accuracy for unrelated stimuli was not affected. A third experiment using a control task where subjects compared pairs of random letters showed no effect of TMS. Taken together these results indicate that the right cerebellum is involved specifically in the processing of semantically related stimuli. These results are consistent with theories that proposed the existence of a unified cerebellar function within motor and non-motor domains, as well with recent perspectives about cerebellar involvement in semantic memory and predictive cognition.

## Introduction

Mounting evidence suggests that the cerebellum, a structure previously linked to motor function, is also involved in a wide range of non-motor processes (e.g., timing, language, working memory; for reviews^1–6^; and for a general discussion see^7,8^). It has been suggested that, across motor and non-motor domains, the cerebellum performs the same one or more key computational processes, allowing motor and cognitive coordination (cognitive coordination can be defined as the regulation of speed, capacity, consistency, and appropriateness of mental or cognitive processes^9^; but see also^10^). Consistent with this perspective, studies showed that the microstructure of the cerebellar cortex is uniform^11^ and that cerebro-cerebellar connections are segregated^12–14^. It is thought that structural uniformity could underlie functional uniformity and the segregated cerebro-cerebellar connections would allow specific cerebellar areas to participate in specific cognitive functions (for a review see^15^).

The cerebellar anatomy and functional properties are well suited for it to be an area in which information from multiple sources converges and is integrated^16,17^. One property of an integration area is that it is expected to be amodal/multimodal^18,19^. Indeed, the cerebellum, particularly the anterior lobe, is thought to integrate proprioceptive, vestibular, visual and motor efference information in order to create a unified, multimodal representation of an event^2^. Similarly, it has been shown that the cerebellum is involved in online integration of sensory and motor information^20^, such as when integrating haptic information from one hand and visual information from a video of the hand^21^, as well as in somatic-visceral^22^ and multisensory integration^23^.

Besides being amodal/multimodal, brain areas involved in integrative processes are proposed to be able to store and use flexible representations of objects, that is for example the concept of “chair” can be used to recognize as chair a *black* chair, a *white* chair or even a chair with wheels, thus allowing flexible use of the stored knowledge^18,19^). The cerebellum indeed is able to store and use flexible representations of sensory-motor internal models of tools^24^. Thus, given the hypothesis of cerebellar functional uniformity across both motor and non-motor domains, one might expect that the cerebellum performs integration functions not only in motor domains but also in other mental activities^25^ such as semantic memory.

Here we hypothesized that cerebellum may be involved in integration within semantic memory, that is in the combination of multiple concepts in order to represent more complex meanings, such as when integrating the meaning of two words (“red” and “car”) in one more complex representation (i.e., “red car”). Consistent with this, (right posterior) cerebellar activations have been reported in combinatorial semantic processing^26^, such as when merging the meaning of two nouns in one more complex concept. Furthermore, neuroimaging studies showed that the (right) cerebellum is involved in a wide range of semantic and language processes that could underlie semantic integrative processes (e.g., reading^27^; semantic memory^28^; lexical processing^29^; object naming^30^; and language production^31^; for a review^32^). One reason why a link between cerebellum and semantic integrative processes has not been established previously is that impairments in semantic function are not a widely recognized symptom in patients with (right) cerebellar lesions. One study did find that patients with cerebellar lesions were impaired in higher level linguistic abilities, such as pragmatics or context adaptation, which possibly may be due deficits in semantic integration^34^. However, to date no causal evidence of cerebellar involvement in semantic integration is available.

To test whether cerebellum is causally involved in semantic integration, we performed three experiments using Transcranial Magnetic Stimulation (TMS). In two experiments, we asked participants to judge if noun-adjective word-pairs were semantically related or not (e.g., red apple vs. lucky milk, respectively) while TMS was administered. This task requires accessing and retrieving from semantic memory the meaning of the words and then to judge if they are semantically compatible or not. In two separate sessions, TMS was administered over the right cerebellum and over a control area (vertex in Experiment 1, visual cortex in Experiment 2). To assess whether our results were selective for semantic memory, we also performed a third experiment using a control task where subjects compared random letter strings. TMS was administered over the right posterior cerebellar hemisphere because, firstly, language and semantic processes are right-lateralized in the cerebellum^32^; and secondly, posterior cerebellar lobes are involved in non-motor functions (for a review^10,11^). We hypothesized that a specific role for the cerebellum within semantic memory would result in an impairment in accuracy only for related words (where semantic integration is possible), while a more general role for cerebellum in reading or phonological processes would result in a decrease in accuracy for both related and unrelated word-pairs.

## Experiment 1

### Methods

#### Power analysis

The minimum sample size was estimated through G*Power^33^ using as effect size *ηp*^2^ = 0.20, *α* = 0.05, 1 − *β* = 0.95 and correlation among repeated measurements *r* = 0.50. The effect size estimation was performed following experimental evidence reported by TMS studies targeting the right cerebellum^35^. The minimum sample size was 16.

#### Participants

Twenty-four students in the psychology program at University of Pavia (4 males; mean age = 23.4 years, SD = 2.4) participated in the experiment. All participants were native Italian speakers, right-handed (^36^; all participants had laterality quotient > 50) and had normal or corrected to normal vision. Prior to the experiment, each participant provided written informed consent and filled out a questionnaire (translated and adapted from^38^) to evaluate their eligibility for the TMS experiment. None of the participants reported neurological problems or was taking medication that could interfere with neuronal excitability. The protocol was approved by the ethical committee of the University of Pavia and participants were treated in accordance with the Declaration of Helsinki.

#### Stimuli

We used the word-pair task introduced by Price, Peelle, Bonner, Grossman and Hamilton^37^. Participants were shown a noun-adjective pair whose ﻿combination was considered to be either semantically related (e.g. red apple) or unrelated (e.g. lucky milk) and were asked to judge if the noun-adjective association was related or not. The relatedness of the word-pairs ﻿was determined by a pilot study (*N* = 23), all the nouns were concrete (words in the range between 500 and 700; *M* concreteness = 652, *SD* = 47.81; maximum value of concreteness = 700) and were taken from an Italian database^39^.

We created two sets of stimuli (labeled “set A” and “set B”) of word-pairs matched for the reaction times (RTs) data collected, on the logarithm of the frequency of the word-pair in CoLFIS (Corpus and Frequency Lexicon of Written Italian^40^), and on the length of the word-pair (Table 1). The processing of related word-pairs required shorter RTs (*p* < 0.001), as previously reported^41^.

**Table 1:** Means and standard deviations of the variables used to match the word-pairs of the two sessions employed in Experiment 1 and Experiment 2.

	RTs	BF	Log frequency	BF	Length	BF
Session A	1250 ms (178)	5.13	0.34 (0.63)	5.14	14.10 (1.67)	4.94
Session B	1249 ms (163)		0.34 (0.56)		14.01 (1.40)	
Related A	1135 ms (117)	3.81	0.68 (0.75)	3.81	13.96 (2.32)	3.80
Related B	1134 ms (113)		0.68 (0.63)		13.93 (1.91)	
Unrelated A	1365 ms (153)	3.80	1.77* (0.53)	3.57	14.23 (0.50)	2.66
Unrelated B	1363 ms (120)		1.67* (0.60)		14.10 (0.60)	
Related	1134 ms (114)	< 0.01	1.81* (0.62)	3.51	13.95 (2.11)	3.93
Unrelated	1364 ms (137)		1.72* (0.56)		14.16 (.55)	

Bayes Factors (BFs) were computed using JASP in its default settings for the a priori distribution of the parameters (Cauchy distribution, located at 0, scale = 0.707^45^). In the present analysis, BFs above 1 indicate evidence for the null hypothesis and BF below 1 indicate evidence for the alternative. We considered BFs above 3 indicative of moderate evidence in favor of the null hypothesis^42^. Asterisks indicate that the reported data are relative to the frequency of the words that composed each condition/session taken alone and not within a word pair (unrelated word-pairs have no frequency by definition).

In order to test if the two sets were matched correctly, we carried out a second pilot study (*N* = 16), in which participants were shown the two sets of word-pairs and were required to judge if the noun-adjective was related or not. As in our prior pilot study, we found that semantically related word-pairs required shorter RTs compared with semantically unrelated word-pairs (*p* < 0.001, *ηp*^2^ = 0.83). No differences were found in accuracy or RTs across sessions (all *p* values > 0.60, all *ηp*^2^s < 0.02). Since we were interested in estimating the relative evidence supporting the null versus the alternative hypothesis^42^, we performed two Bayesian repeated-measures ANOVAs using JASP in its default settings for the a priori distribution of the parameters (r scale fixed effects = 0.5, r scale random effects = 1; for more information regarding priors see^43–45^) and adding the type of stimuli (related vs. unrelated) in the null model. Since the Bayes Factor (BF) computation is a ratio between the probabilities of two different hypotheses, in the present analysis BFs above 1 indicate evidence for the null hypothesis and BF below 1 indicate evidence for the alternative. We considered BFs above 3 indicative of moderate evidence in favor of the null hypothesis^42^. The BFs of the null models were both > 5.30.

Due to the small effect sizes of the differences and BFs, we concluded that the two sets of stimuli were equivalent. During the main experiment, these two sets of stimuli were presented in two separate blocks, with their order counterbalanced across participants (see details below).

#### Procedure

Participants were seated comfortably at a distance of 60 cm from a 17″ computer monitor with their head stabilized using a chinrest during the stimulation. Participants were shown the two blocks of word-pairs and they were required to judge if the noun-adjective was semantically related or not. Stimuli were displayed on a computer monitor using Matlab (Mathworks, Inc.) and the Psychophysics Toolbox extensions^46–48^. Participants were instructed to respond as fast and as accurately as possible by pressing the left/right key (J and K) pressing using their right hand; the response keys were counterbalanced across participants. The trials were shown in random order.

**Figure 1:**
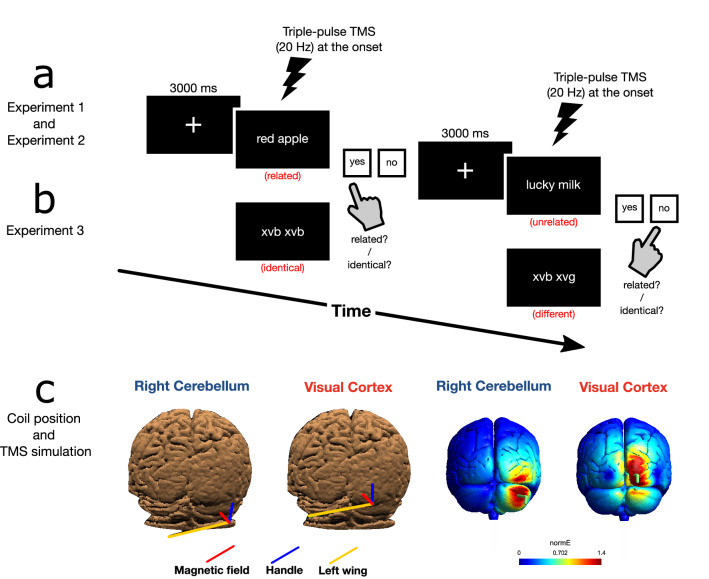
Methods. In Experiment 1 and 2, participants were asked to judge whether the word-pairs presented were semantically related (e.g., red apple) or unrelated (e.g., lucky milk) while TMS was delivered (**a**). In Experiment 3, participants were asked to judge whether random-letters pairs were identical or not while TMS was delivered (**b**). On each trial a stimulus was presented while a train of TMS pulses was delivered and the trial ended when participants responded by button press. Right cerebellum (left) or visual cortex (right) were selected as target sites for TMS as well as the vertex as control site (not shown), blue indicates the handle orientation, yellow the left-wing of the coil and red the magnetic field generated by the coil on a 3D-rendered sample T1 obtained using Softaxic (Softaxic 2.0, EMS, Bologna, Italy) (**c**). On the right is reported the estimated electric field induced by TMS Magstim 70 mm figure-of-eight coil obtained using SimNIBS^94,95^. Warmer colors indicate a stronger electric field, in green is reported the direction of the magnetic field (the line perpendicular to the cortex) and the direction of the coil (the side of the coil opposite to the handle).

On each trial, a central fixation cross was presented for 3000 ms followed by a word-pair, which remained on the screen until participants pressed the response key at which point the trial ended (see Fig. 1a).

TMS was delivered at the onset of the word-pair stimulus. The experiment consisted of two experimental blocks (each block consisted of 60 stimuli, 30 meaningful and 30 non-meaningful, see above), one for each TMS site (TMS over the right cerebellum, TMS over the vertex). A short practice session was presented at the beginning of the experiment to familiarize participants with the task. Order of TMS sites and blocks was counterbalanced across participants.

#### Transcranial magnetic stimulation (TMS)

Online neuronavigated TMS was performed with a Magstim Rapid^2^ stimulator (Magstim Co., Ltd, Whitland, UK) and a 70 mm figure-of-eight coil. Individual motor threshold (MT) was determined at the beginning of each session using single pulse TMS at increasing intensities. MT is defined as the lowest stimulation intensity delivered over the left motor cortex able to evoke a muscle twitch in the contralateral hand in 5/10 consecutive trials (for methodological details see^49^). During the experiment, participants were stimulated at 100% of their MT; Mean TMS intensity delivered: 48.1% of the maximum stimulator output, *SD* = 3.3%. Triple-pulse 20 Hz TMS was delivered at the onset of each stimulus.

TMS was delivered over the right cerebellum and the vertex (control condition) (see Fig. 1c). The cerebellum was localized by means of stereotaxic navigation on individual estimated magnetic resonance images (MRI) obtained through a 3D warping procedure fitting a high-resolution anatomical MRI T1 template with the participant’s scalp model and craniometric points (Softaxic, EMS, Bologna, Italy). MNI coordinates used for neuronavigation were x = 32, y =  − 74, z =  − 37 for the right cerebellum, corresponding to Crus I/Crus II, cerebellar loci of activation reported in a previous neuroimaging study investigating semantic prediction^50^. The estimated distance between coil focus and cerebellar cortex was ~ 15 mm^51^ and the coil used was shown to stimulate reliably up to 30 mm of depth^52^ and posterior cerebellar areas^53^. The vertex was localized as the point falling half the distance between the nasion and the inion. The coil was placed tangentially to the scalp with the handle pointing superiorly during the stimulation over the right cerebellum (see Fig. 1c) and with the handle pointing backward during the stimulation over the vertex. Since repetitive TMS over posterior cerebellar areas can induce muscular twitches, prior to the experiment a few TMS pulses over the right cerebellar hemisphere were administered in order to familiarize participants with the skin sensations. This method of stimulation has been adopted successfully previously in TMS experiments targeting the cerebellum (e.g.^35,54–58^).

#### Data analysis

In order to assess whether cerebellar TMS had a disruptive effect on participants’ performance when judging the relatedness of the word-pairs showed, we analyzed mean accuracy through a 2 × 2 ANOVA with TMS site (right cerebellum vs. vertex) and relatedness (related word-pairs vs. unrelated word-pairs) as within-subject factors. In case of significant difference between two measures, we also performed a linear regression to test if the difference in mean accuracy was predicted by a difference in median RTs (i.e., caused by a speed-accuracy tradeoff). Median correct RT data was analyzed through a 2 × 2 ANOVA with TMS site (right cerebellum vs. vertex) and relatedness (related word-pairs vs. unrelated word-pairs) as within-subject factors. RTs were analyzed using the median as a measure of central tendency because RT distribution are typically skewed.

### Results

Participants were asked to judge whether noun-adjective pairs were semantically related or not while TMS was delivered over the right cerebellum or the vertex. The main dependent variable was response accuracy; as a control measure we also collected correct reaction times (RTs) for each participant in each block. Trials in which participants’ reaction times (RTs) were further than 3 *SD* away from participant’s block mean were excluded from the analyses (0.9% of trials were excluded).

**Figure 2:**
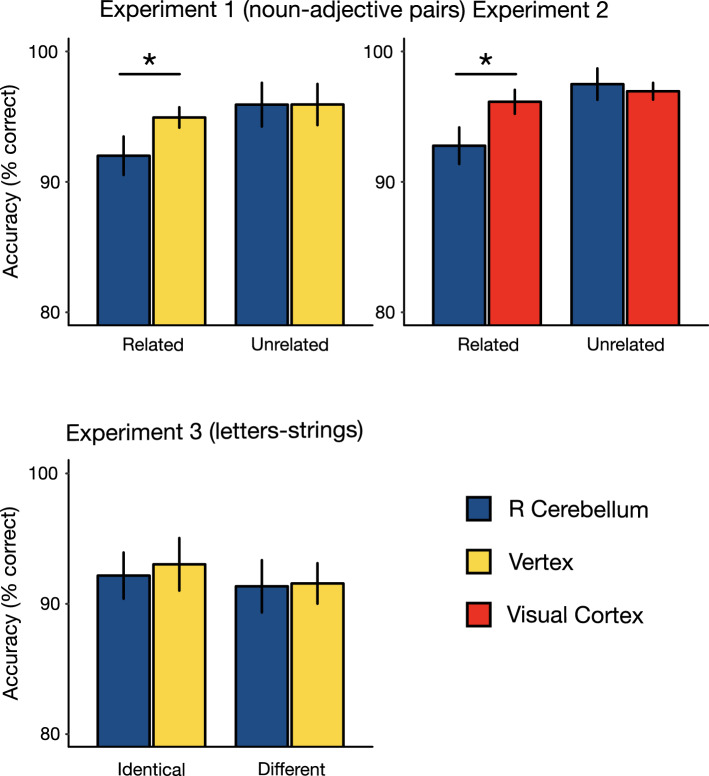
Results. In Experiment 1 and 2, during cerebellar TMS participants were less accurate recognizing semantically related noun-adjective word-pairs compared with control conditions. This effect was not observed for unrelated word-pairs or for control site stimulation (vertex in Experiment 1 or visual cortex in Experiment 2). In Experiment 3, no differences were found in participants’ accuracy between the two TMS sites during the random-letters task. Mean percentage accuracy scores are shown; error bars represent ± Standard Error of the Mean; asterisks indicate significant differences between conditions (*p < 0.05).

In order to assess if TMS affected response accuracy we performed an ANOVA that revealed a main effect of TMS site, *F*(1,23) = 4.15, *p* = 0.05, *ηp*^2^ = 0.15, indicating that participants’ accuracy was lower during cerebellar TMS compared with vertex stimulation, and a statistical trend towards a main effect of relatedness, *F*(1,23) = 3.63, *p* = 0.06, *ηp*^2^ = 0.13, showing that participants’ accuracy was lower for related word-pairs compared with unrelated word-pairs; critically, the interaction TMS site by relatedness was significant, *F*(1,23) = 5.26, *p* = 0.03, *ηp*^2^ = 0.18. Planned contrasts revealed that, for related word-pairs, participants’ accuracy was lower during right cerebellar TMS (Prop. correct = 0.92, *SE* = 0.01) compared with TMS over the vertex (Prop. correct = 0.95, *SE* = 0.008), *t*(23) = − 3.04, *p* = 0.004; whereas no difference in accuracy for unrelated word-pairs were found between the two TMS conditions (Prop. correct right cerebellum = 0.96, *SE* = 0.01; Prop. correct vertex = 0.96, *SE* = 0.01), *t*(23) =  − 0.009, *p* = 0.99 (see Fig. 2—Experiment 1).

We also analyzed reaction times (RTs): the ANOVA on the median RTs revealed a trending main effect of TMS site, *F*(1,23) = 3.52, *p* = 0.07, *ηp*^2^ = 0.13, showing that participants’ RTs were faster during cerebellar stimulation (median RTs = 1076 ms, *SE* = 23 ms) compared with vertex stimulation (median RTs vertex = 1107 ms, *SE* = 26 ms) and a main effect of relatedness, *F*(1,23) = 42.22, *p* < 0.001, *ηp*^2^ = 0.64, indicating that RTs for related word-pairs were faster (median RTs = 1044 ms, *SE* = 22 ms) compared with unrelated word-pairs (median RTs = 1139 ms, *SE* = 25 ms), but no interaction TMS site by relatedness effect was found, *F*(1,23) = 0.02, *p* = 0.88, *ηp*^2^ = 0.001.

These analyses revealed that participants’ accuracy was lower during right cerebellar TMS compared with TMS over the vertex, moreover RTs analysis revealed a non-significant trend for responses to be faster during cerebellar TMS. To exclude the possibility that the participants with lower accuracy were also those with faster RTs and thus to assess if the decrease in accuracy was induced by the TMS or was explained by the decrease in RTs (i.e., speed-accuracy tradeoff), we performed a linear regression on the differences of mean accuracy and of median RTs during the two TMS conditions. The decrease of accuracy for meaningful word-pairs was not explained by a difference in RTs, *r* = − 0.25,* p* = 0.22, ruling out speed-accuracy tradeoff (see Supplementary material).

## Experiment 2

In Experiment 1, TMS selectively affected semantically related word-pairs processing without affecting non-meaningful word-pairs. This result ruled out the possibility that the detrimental effect of TMS depended on unspecific effects of the stimulation. However, in principle it is possible that the accuracy reduction during cerebellar TMS is due to indirect stimulation over the visual cortex^59^. Indeed, it has been shown that cerebellar TMS induces “widespread supra-threshold electric fields in the occipital lobe of the cerebral cortex” and it has been argued that studies administering TMS over the cerebellum should control for indirect stimulation of visual areas (^53^ p. 678). In the present work simulations suggested that the visual cortex may have been stimulated during cerebellar TMS (Fig. 1c). Although these are simulations and not empirical observations, we wanted to ensure that the effects observed in Experiment 1 were due to the stimulation of the cerebellum, not inadvertent occipital stimulation. We thus ran a control experiment where we compared occipital cortex and cerebellar stimulation directly. If potential occipital cortex spillover stimulation during cerebellar TMS lead to our findings, we should observe the same behavioral pattern of results across cerebellar and occipital stimulation. Alternatively, if the effect observed in Experiment 1 is due to cerebellar stimulation proper, we expect to see semantic integration impairment only in the cerebellar TMS condition.

### Methods

The stimuli, procedure and data analysis were identical to Experiment 1.

#### Power analysis

Power analysis was identical to Experiment 1 and revealed a minimum sample size of 16 persons.

#### Participants

Twenty students in the psychology program at University of Pavia (3 males; mean age = 21.7 years, *SD* = 1.08) participated in the experiment. Inclusion criteria were identical to Experiment 1. None of the participants had participated in the previous experiments.

#### Transcranial magnetic stimulation (TMS)

TMS procedures were identical to Experiment 1; the only difference was that as control condition, instead of the vertex, we administered the stimulation over the right primary visual cortex (see Fig. 2c). MNI coordinates used for right primary visual cortex were x = 17, y =  − 98, z =  − 10 (^60^ but see also^61^). The coil was placed tangentially to the scalp with the handle pointing superiorly both during the stimulation over the right cerebellum and over the right primary visual cortex. Mean TMS intensity delivered: 47.4% of the maximum stimulator output, *SD* = 3.1%.

### Results

Participants were asked to judge whether noun-adjective pairs were semantically related or not while TMS was delivered over the right cerebellum or the primary visual cortex. The main dependent variable was response accuracy; we also collected correct reaction times (RTs) for each participant in each block. Outliers were discarded according to the same criteria as in Experiment 1 (1.2% of trials were excluded).

As in Experiment 1, we found that the interaction of TMS site by relatedness was significant, *F*(1,19) = 9.52, *p* = 0.006, *ηp*^2^ = 0.33. For related word-pairs, participants’ accuracy was lower during right cerebellar TMS (Prop. correct = 0.92, *SE* = 0.01) compared with TMS over the primary visual cortex (Prop. correct = 0.96, *SE* = 0.01), *t*(19) = − 2.59, *p* = 0.01; whereas no difference in accuracy for unrelated word-pairs was found between the two TMS conditions (Prop. correct right cerebellum = 0.97, *SE* = 0.01; Prop. correct visual cortex = 0.96, *SE* = 0.006), *t*(19) = 0.42, *p* = 0.67 (see Fig. 2). ANOVA on the mean accuracy also revealed a significant effect of relatedness, *F*(1,19) = 9.49, *p* = 0.006, *ηp*^2^ = 0.33, showing that participants’ accuracy was lower for related word-pairs compared with unrelated word-pairs, but not of TMS site, *F*(1,19) = 1.55, *p* = 0.22, *ηp*^2^ = 0.07.

We also analyzed RTs. ANOVA on the median RTs revealed a main effect of relatedness, *F*(1,19) = 79.63, *p* < 0.001, *ηp*^2^ = 0.80, indicating that participants RTs were faster for related word-pairs (median RTs = 1054 ms, *SE* = 19 ms) compared with unrelated word-pairs (median RTs = 1183 ms, *SE* = 22 ms), but not of TMS, *F*(1,19) = 0.82, *p* = 0.37, *ηp*^2^ = 0.04, or of the interaction TMS by relatedness, *F*(1,19) = 2.49, *p* = 0.13, *ηp*^2^ = 0.11.

The analysis on accuracy revealed that participants’ accuracy was lower during right cerebellar TMS compared with TMS over the visual cortex. However, despite RTs analysis showed that RTs for related and unrelated word-pairs were not effectively modulated by the TMS, participants’ RTs for related word-pairs were slightly faster during cerebellar TMS (median RTs = 1038 ms, *SE* = 25 ms) compared with primary visual cortex (median RTs = 1070 ms, *SE* = 30 ms). On the contrary, participants’ RTs for unrelated word-pairs were comparable across the two TMS conditions (median RTs right cerebellum = 1183 ms, *SE* = 32 ms; median RTs primary visual cortex = 1184 ms, *SE* = 32 ms). As in Experiment 1, to exclude the possibility that the participants with lower accuracy were also those with faster RTs and thus to assess if the decrease in accuracy was explained by the decrease in RTs, we performed a linear regression on the differences of mean accuracy and of median RTs during the two TMS conditions. The decrease of accuracy for meaningful word-pairs was not explained by a difference in RTs, *r* = − 0.04,* p* = 0.84, ruling out speed-accuracy tradeoff (see Supplementary material).

## Experiment 3

In Experiments 1 and 2, cerebellar TMS selectively affected semantically meaningful word-pairs processing without affecting unrelated word-pairs. These results ruled out the possibility that the detrimental effect of TMS depended on unspecific effects of the stimulation and further analysis on RTs ruled out the speed-accuracy tradeoff. However, in order to test whether the right cerebellum is involved specifically in semantic processing or in lower level processes such as word reading, we carried out a third experiment in which we used the same TMS procedures previously used in Experiment 1 while participants were asked to perform a control task. If cerebellar TMS specifically affects semantic processing this experiment should reveal no differences between the TMS conditions.

### Methods

#### Power analysis

Power analysis was identical to Experiment 1.

#### Participants

Twenty-one students in the psychology program at University of Pavia (4 males, mean age = 23.3 years, *SD* = 2.6) participated in the experiment. Inclusion criteria were identical to Experiments 1 and 2.

#### Stimuli

We used the random-letters task introduced by Price and colleagues^37^. Participants were shown two strings of letters that could be either identical or differ in one letter and participants were asked to judge if the two strings were identical or not. The stimuli were constructed through a random letter generator (https://www.dave-reed.com/Nifty/randSeq.html) using only consonants. The letter that caused the difference between two stimuli and its position were chosen randomly.

Price and colleagues^37^ used as stimuli strings of five letters (e.g., xvbhr xvbhr, xvbhr xvghr). Here, in order to use stimuli with RTs comparable with those of Experiment 1 and 2 we used as stimuli strings of three letters (e.g., xvb xvb, xvb xvg). A pilot study (*N* = 16) was carried out in order to collect RTs data (median RTs = 1079 ms, *SE* = 49 ms).

#### Transcranial magnetic stimulation (TMS)

TMS procedures were identical to Experiment 1. The TMS sites were right cerebellum and vertex. Mean TMS intensity delivered: 48.8% of the maximum stimulator output, *SD* = 1.9%.

#### Procedure

Procedure was identical to Experiment 1; the only difference was that participants were shown pairs of random letters and asked to judge if the two stimuli were identical or not.

TMS was delivered at the onset of the random-letters pair. The experiment consisted of two experimental blocks (each block consisted of 60 stimuli, 30 identical and 30 different), one for each TMS site (TMS over the right cerebellum, TMS over the vertex). A short practice session was presented at the beginning of the experiment to familiarize participants with the task. Order of TMS sites and blocks was counterbalanced across participants.

#### Data analysis

Data analysis was identical to Experiment 1; the within-subject factor was the type of stimulus (identical vs. different). Since we were interested in estimating the relative evidence supporting the null versus the alternative hypothesis^42^, we also performed two Bayesian repeated-measures ANOVAs using JASP in its default settings for the a priori distribution of the parameters (r scale fixed effects = 0.5, r scale random effects = 1; for more information regarding priors see^43–45^) and adding the type of stimuli in the null model. Since BF computation is a ratio between the probabilities of two different hypotheses, in the present analysis BFs above 1 indicate evidence for the null hypothesis and BF below 1 indicate evidence for the alternative. We considered BFs above 3 indicative of moderate evidence in favor of the null hypothesis^42^.

### Results

Participants were asked to judge whether pairs of random letters were identical or not while TMS was delivered over cerebellum or the vertex. The main dependent variable was response accuracy; we also collected correct reaction times (RTs) for each participant in each block. Outliers were discarded according to the same criteria as in Experiments 1 and 2 (1% of trials were excluded).

In order to assess if TMS affected response accuracy we performed an ANOVA that showed that participants’ accuracy was not modulated by the site of TMS, *F*(1,20) = 0.30, *p* = 0.58, *ηp*^2^ = 0.01, nor by the type of stimulus, *F*(1,20) = 2.09, *p* = 0.16, *ηp*^2^ = 0.09; similarly the interaction TMS site by type of stimulus was not significant, *F*(1,20) = 0.19, *p* = 0.66, *ηp*^2^ = 0.01. The BF of the null model was 5.45, indicating moderate evidence in favour of there being no difference between cerebellar and vertex TMS. Participants’ accuracy for both identical stimuli (Prop. correct right cerebellum = 0.92, *SE* = 0.01; Prop. correct vertex = 0.93, *SE* = 0.02) and different stimuli (Prop. correct right cerebellum = 0.91, *SE* = 0.02; Prop. correct vertex = 0.91, *SE* = 0.01) was not significantly modulated by TMS (see Fig. 2).

As in our previous experiments, we also analyzed RTs as control analysis. The ANOVA on the median RTs revealed that participants’ RTs were not modulated by the site of TMS, *F*(1,20) = 0.25, *p* = 0.61, *ηp*^2^ = 0.01; regardless of the TMS effect, participants’ RTs were faster for different stimuli (median RTs = 964 ms, *SE* = 27 ms) compared with identical stimuli (median RTs = 1020 ms, *SE* = 31 ms), *F*(1,20) = 16.06, *p* < 0.001, *ηp*^2^ = 0.44. The interaction TMS site by type of stimulus was not significant, *F*(1,20) = 0.15, *p* = 0.70, *ηp*^2^ = 0.008. The BF of the null model was 5.62, indicating moderate evidence in favour of there being no difference between cerebellar and vertex TMS.

Thus, we can conclude that cerebellar TMS did not modulate participants’ performance during the pairs of random letters task.

## Discussion

The present study tested the hypothesis that the cerebellum is involved in integrating information in semantic memory. Participants were asked to judge if a noun and adjective were semantically related or not while TMS was administered over the right cerebellum or over a control site (vertex or visual cortex). Right cerebellar TMS caused a decrease in participants’ accuracy for semantically related word-pairs; conversely accuracy for unrelated word-pairs was not affected. The disruptive effect of cerebellar TMS was present in Experiment 1 and replicated with a different control area of stimulation (Experiment 2). Cerebellar TMS only affected accuracy for related word-pairs without affecting accuracy for unrelated ones, suggesting that cerebellar TMS specifically disrupted semantic memory integration, instead of affecting the processing of individual words. Furthermore, the selectivity of TMS effects for related word-pairs ruled out unspecific effects of stimulation, and the results of Experiment 2 verified that the observed effect was not due to indirect stimulation of the visual cortex^59^. Additional analyses ruled out a potential speed-accuracy trade-off, showing that participants with lower accuracy did not exhibit faster RTs. In Experiment 3 we used the same TMS procedure used in Experiment 1 while participants performed a control task comparing strings of random letters instead of words and we found that cerebellar TMS did not affect participants’ performance. Results of Experiment 3 suggest that it is unlikely that the accuracy decrease in Experiment 1 and 2 depend on unspecific effects of TMS stimulation on motor control or visual processing. The present results contribute to mounting evidence for cerebellar involvement in semantic memory.

While previous studies have shown cerebellar involvement in integration in sensory and motor domains^20–23^, our study extends this by showing that cerebellum also performs integration in the semantic domain. Our results, taken together with previous studies showing cerebellar involvement in motor and sensory integration, is consistent with the idea that the cerebellum performs the same function in both motor and non-motor domains^9^. In a wider context, since semantic integration processes rely on semantic memory^37^; our results are also consistent with previous evidence^28^ and recent hypotheses about cerebellar participation in semantic memory^62^.

The present finding supports existing theories according to which cerebellum acquires, stores and uses relations between co-occurring linguistic events^63,64^. Neuroimaging, neuromodulation and lesion studies showed that the cerebellum is involved in processing words that are semantically related (e.g., *soap-cleaning*^65,66^) and in generating related verbs for given nouns (e.g., given the word *eat,* generate for example *cake*^29,67–69^. Consistent with this perspective, in the present study cerebellar TMS selectively affected the processing of semantically related word-pairs. The related word-pairs used in our study were nouns and adjectives that frequently co-occur in written and oral speech and thus are likely generally associated in people’s minds. Since the cerebellum is thought to be involved in the processing of words that are generally associated in language^63,64^, the selective drop in participants’ accuracy for related word-pairs would reflect the disruption of the processing of associations, probably impairing the retrieval from semantic memory. The present study extends the previous findings in two ways: first, it shows that the cerebellar involvement in processing words semantically associated in language is causal, not merely correlational; second, it links the cerebellum specifically to semantic integration, not merely semantic association. Guell and colleagues^34^ demonstrated that patients with cerebellar lesions show impaired performance in higher-level linguistic abilities, such as pragmatics or context adaptation. Together with the findings of our study, this raises the possibility that these deficits may arise from impairments in integrative processes, however more research is needed to establish whether that is the case.

Cerebellar participation to semantic integration could be related to direct participation in semantic processing, as well as to indirect modulation^70^ of the cortical brain areas involved in semantic memory and connected to the cerebellum, such as frontal and temporal areas^27,71^. However, the mechanisms underlying cerebellar participation in cognitive processing remain to be clarified. In the case of direct cerebellar participation, the present results may be viewed from the perspective of previous work that investigated cerebellar participation to sensory-motor integration. It is thought that sensory-motor integration in the motor cortex and in the cerebellum occur in different manners: on the cortical level, each movement is paired with the most likely sensory outcome of that movement (hence sensory-motor stimulation activates the same microzones of cortex), while in cerebellar cortex, each movement activates neurons related to a wider range of different sensory outcomes “ranging from stimulation of other fingers to maybe even sensory input from the arm or face” (^20^, p. 3051). This dissociation between cerebellar and cortical levels may be mirrored in semantic processing as well. Previous studies have shown that anodal stimulation over the left angular gyrus results in faster comprehension of semantically meaningful combinations^26^, while the results of our study show that right cerebellar TMS interferes with participants’ accuracy in judging the relatedness of meaningful word-pairs. These differential effects of brain stimulation (i.e., response latencies facilitation vs. accuracy impairment) could reflect the different nature of semantic integration in cortical areas and in the cerebellum. Consistent with this, patients with cerebellar lesions showed increased angular gyrus activation during verbal tasks, suggesting compensatory recruitment to maintain task performance^72^, and adults with developmental dyslexia showed abnormal connectivity between cerebellum and angular gyrus during reading tasks^73,74^. Right cerebellum, and temporo-parietal areas are functionally and anatomically connected^12,75–78^ and may interact in semantic memory processes contributing at different time points or performing different computations at the same time.

Our finding of cerebellar involvement in semantic memory is in line with models of semantic memory relying on fronto-temporo-parieto-cerebellar circuits. According to Ito^25^, frontal areas act as executive controllers on mental objects stored in temporal and parietal cortices; the manipulation of these objects would take place within cerebellar cortices through internal models (for a review on internal models in the cerebellum^79^). In the context of this perspective, the results of previous studies on semantic memory taken together with the results of our study suggest the following account: frontal areas are active during executive control^28^, temporo-parietal areas store long-term semantic memories needed in the word-pair task (which is why transcranial direct current stimulation can facilitate the retrieval, i.e., reducing response latencies of the memories stored^37^), and the cerebellum would allow the manipulation of the objects retrieved from semantic memory. In the present study, the impaired accuracy for the semantically related word-pairs observed during cerebellar TMS would reflect a disruption of the manipulation of the word-pair. In order to judge whether a word pair (e.g. *red* and *apple*) is semantically related or not, the brain would have to retrieve the meanings of the individual words and then attempt to integrate them. Since the object in question is not present (e.g. there is no red apple) this process presumably relies on internal simulation. That is, the cerebellum could provide the semantic features (which are embedded in cognitive internal models, see^25^) of the words needed for judging if a word pair is related. Possibly the cerebellum would run the mental simulation (i.e., the integrative process) of the semantic memories retrieved from temporo-parietal areas. The lack of effect for unrelated word-pairs is consistent with this perspective, because unrelated stimuli are not semantically associated and hence there is no integration to be made.

The present results also advance the cerebellum as a possible neural substrate for the intriguing link between memory and prediction (for a review^62^). Although the present task does not directly measure prediction, presumably prediction is involved when the first word (in Italian the noun generally precedes the adjective) automatically activates the second one, similarly to what happens in associative and semantic priming tasks. Several studies have linked cerebellar activity to predictive functions (for a review^80^), such as sequence detection^81^, internal model processing^17,82^ or timing^83^. It has also been argued that prediction and memory are based on the same cognitive process, namely mental simulation, directed either forward (in the case of prediction) or backward (in the case of memory) in time^84–86^. The cerebellum is a suitable candidate for the neural substrate of both prediction and memory. Indeed, several studies showed that the cerebellum is part of a wide network engaged during both retrieval and future thinking^87,88^. Our results, taken together with previous studies that showed that the right cerebellum is involved in semantic prediction^50,89^, might corroborate neuroimaging evidence about cerebellar participation in both memory retrieval and predictive processes in semantic domains. However, the link between the present task and prediction remains speculative. A future study could aim to clarify this link by manipulating the timing of TMS (at the onset of the first word vs. at the onset of the second, possibly dividing the two words in two different screens). In case of cerebellar involvement in predictive functioning we would expect TMS to affect participants’ performance when delivered at the same time as the first word (i.e., when the prediction is made) rather than when it is delivered during the second word (i.e., when the integration is made).

More studies are needed to clarify cerebellar involvement in semantic memory and cognitive functioning in general. Future studies might focus on lateralization^90^ and asymmetries (e.g., verbal vs. non-verbal^91,92^) of memory processes performed by the cerebellum as well as investigate cerebellar involvement in semantic memory from a chronometric point of view.

The present study has several limitations. The a priori distinction in semantically related and unrelated word pairs was based on a database employing data from written language and thus could be biased relative to spoken language, since oral language word frequencies could be different from written language, resulting in different results of the analyses performed on the stimuli employed. Similarly, the dichotomy in semantically related and unrelated is in some cases arbitrary; future studies could address this issue by using a continuous value of semantic relatedness measured empirically^37^ or calculated through distributional semantic models (for a review^93^). In the random letter string control task we found no effect of cerebellar TMS; a limitation of this finding is that, although used in previous studies^37^, the letter strings are shorter than the word pairs used in the other experiments and the strings do not follow the phonological rules that words do. Future studies could employ readable letter strings, control for the length of the stimuli. The detrimental effect of TMS could also have been caused by the discomfort that participants might have experienced during cerebellar TMS, leading to higher rejection rate for less common related word-pairs. Nevertheless, in that case one might have expected a similar detrimental effect in the random letter strings in Experiment 3 (possibly for those stimuli slightly different for which, in case of discomfort, participants would have failed to recognize the difference), which we did not observe. Furthermore, studies have suggested that discomfort using the figure-of-eight coil is minimal^51^. Finally, participants’ decrease in accuracy was small (around 3%), although similar in magnitude to the effect size reported by previous studies targeting the right cerebellum using triple-pulse stimulation^35^.

In conclusion, our findings indicate that the right cerebellum is causally involved in integrating semantically related words. One possibility that is consistent with these results, is that the cerebellum performs a unified function in motor and non-motor domains, and similarly it is possible that cerebellum is involved in memory and predictive cognition.

## Supplementary information

Supplementary Information.

## Data Availability

All data collected in this experiment are available upon request.
