# Supplementary material for: A causal role for the cerebellum in semantic integration: a transcranial magnetic stimulation study
Source: Sci Rep. 2020 Oct 23;10:18139. doi: 10.1038/s41598-020-75287-z (PMC7584601; doi:10.1038/s41598-020-75287-z)
Supplement: Supplementary file 1 — Supplementary Information. [file 41598_2020_75287_MOESM1_ESM.pdf]

A causal role for the cerebellum in semantic integration: a transcranial magnetic stimulation study

Daniele Gatti [1], Floris Van Vugt [1,2], & Tomaso Vecchi [1,3*]

1 - University of Pavia, Department of Brain and Behavioral Sciences, Pavia, 27100, Italy.

2 - University of Montreal, Psychology Department, H3A1G1, Canada.

3 - IRCCS Mondino Foundation, Pavia, 27100, Italy.

* Corresponding author:

Tomaso Vecchi, Department of Brain and Behavioural Science, University of Pavia, via Bassi 21, 27100 Pavia, Italy. *e-mail address*: vecchi@unipv.it

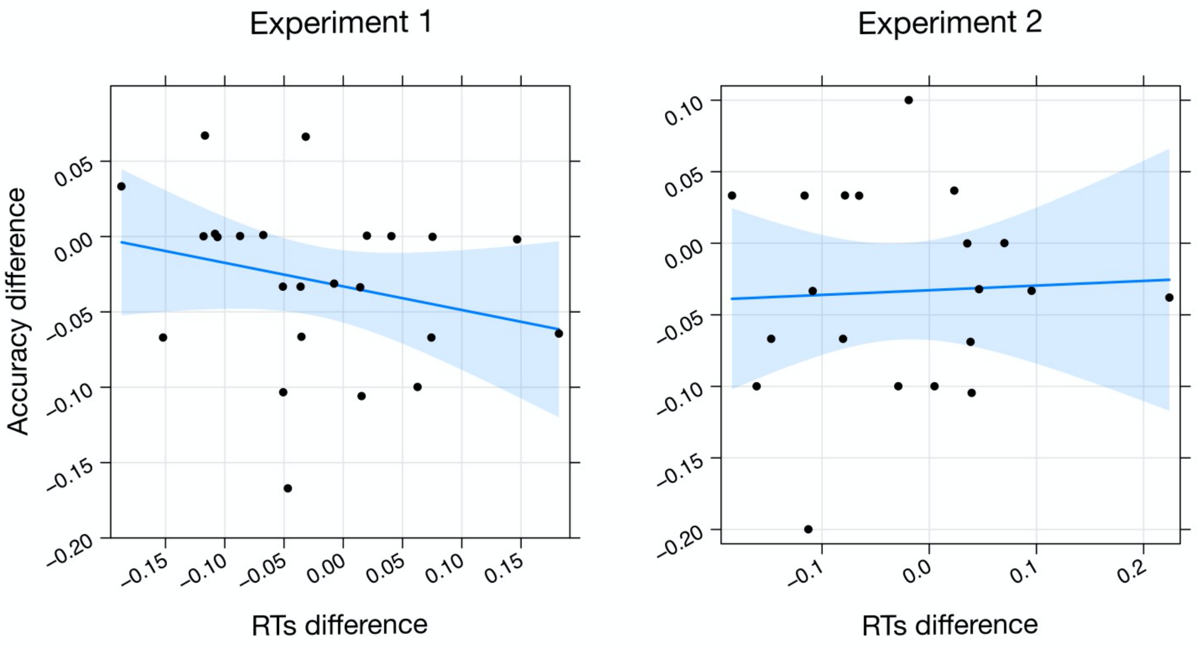

Accuracy difference refers to difference (in proportion) of accuracy for related word-pairs during cerebellar TMS minus during vertex (Experiment 1) or V1 (Experiment 2) stimulation. RTs difference refers to the difference (in seconds) of median RTs for related word-pairs during cerebellar TMS minus during vertex (Experiment 1) or V1 (Experiment 2) stimulation.
